# Optimum Placement of Distribution Generation Units in Power System with Fault Current Limiters Using Improved Coyote Optimization Algorithm

**DOI:** 10.3390/e23060655

**Published:** 2021-05-24

**Authors:** Hisham Alghamdi

**Affiliations:** Electrical Engineering Department, College of Engineering, Najran University, Najran 61441, Saudia Arabia; hg@nu.edu.sa

**Keywords:** fault current limiters, improved coyote optimize algorithm, distribution generation units, main power grids

## Abstract

Electric power frameworks become intensely loaded because of the expanded power demand, and as a result, the power system faces great power losses and fault currents. The integration of Distribution Generation (DG) units plays a key role in minimizing the load pressure on a power system. DGs are transmitted with a high fault current, which surpasses the evaluations of circuit breakers. This paper presents various DG units’ optimal placement with Fault Current Limiters (FCLs) in different phases. The Improved Coyote Optimize Algorithm (ICOA) and Electrical Transient Analyzer Program (ETAP) are assessed for the proposed technique in terms of normal and faulty working status. Similarly, to enhance the efficiency of a distribution system, a fuzzy-based multi-objective mechanism is applied. The proposed method is employed on an IEEE 21-bus and 28-bus distribution system. The simulation analysis proved that the power losses and fault levels are reduced at an acceptable level.

## 1. Introduction

There has been an increase in power demand in the last decade, which means electric grids are heavily loaded [1,2,3]. This continuous pressure of load degrades the overall performance of the main power system. Thus, an economical and technical solution is required to handle load demands. Distribution Generations (DGs) are considered a fruitful solution to minimize the load pressure on a power system economically and technically because DGs improve system fidelity without the installation of new power plants and transmission lines [4,5,6]. However, optimum placement of DGs is a key issue, owing to the wrong allocation of DG units that may generate extra impairments in the power system. Therefore, this work studies the proper placement of DG units in the power system to decrease the power losses and fault current levels.

### Related Work

In order to resolve the proper allocation problem of DGs and reduce power losses and fault current levels, a number of research works have been done so far. In [7], the authors have studied Improved Grey Wolf Optimizer (GWO) for proper placement and sizing of DGs in the distribution system. Another methodology called the genetic algorithm (GA) is presented in [8] to properly allocate DGs in a distribution network for decreasing power loss and enhancing the voltage profile. To minimize power losses and maintain the voltage profile, the Particle Swarm Optimization (PSO) mechanism is analyzed in [9]. Authors have also focused on optimal placement and sizing of DGs in the power system in [9]. In [10], the authors have explored Fault Current Limiters (FCLs) and proper allocation of DGs using a sensitivity factor procedure. A dynamic programming method is presented in [11] for placing and sizing DGs aiming to degrade network loss and improve the voltage profile. In [12], the authors have investigated the optimum sizing and positioning of DGs in the power system by applying the Bacterial Foraging Optimization Algorithm (BFOA). In addition, the GA mechanism is proposed in [13] to save costs and achieve a setting position and FCLs in the distribution system. In [14], the authors have investigated the allocation of various types of DGs integrated with FCLs for a single stage, utilizing COA and an Electrical Transient Analyzer Program (ETAP) for general and faulty states. In [1], the authors have discussed optimum placement of FCLs to minimize fault current levels. The authors of [15] investigate the superlative positions and sizes of single as well as multiple stages of various renewable Distributed Energy Resources (DER) through a hybrid technique built with a Slap Swarm Algorithm (SSA) in addition to combined power loss sensitivity.

However, the presented research models contain several technical and economical issues. Hence, to minimize the power losses and fault current in an installed power distribution system, a new procedure is needed. Thus, this paper studies the optimum placement of DGs and FCLs in a single-phase.

The remaining part of this paper is organized as follows. The analytical model is discussed in Section 2. Section 3 contains the methodology for the proper allocation of DGs and FCLs. Section 4 presents results and discussion, while the conclusion of the proposed work is concluded in Section 5.

## 2. Analytical Modeling

The major purpose of this work is to reduce the power losses of the power system and can be defined [8,16] as

(1) $$g_{1}=min\Sigma_{l=1}^{ik}r_{l}*{\left|I_{l}\right|}^{2},$$

where $r_{l}$ is the resistance, $I_{l}$ presents the current of the distribution lines and ik shows total branches. Decreasing the fault current levels is a second important goal, which is measured [13,17] as

(2) $$g_{2}=min\left(I_{f}\right).$$

Here, $I_{f}$ denotes a three-phase short circuit current. The economical design of FCLs is the third main purpose and is written [18,19] as

(3) $$g_{3}=min\Sigma_{n=1}^{N}SFCL_{n},$$

where SFCL describes the size of the FCL applied, while the total number of FCLs is denoted by N. Furthermore, the constraints of balancing active and reactive powers [20,21,22,23] are analyzed as

(4) $$\begin{array}{c} \beta_{PS}+\sum_{n=1}^{N_{DGUs}}\beta_{n}-\sum_{n_{b}=1}^{N_{b}}PL_{b}=\sum_{n=1}^{N_{bus}}\beta_{d}, \end{array}$$

(5) $$\begin{array}{c} \zeta_{PS}+\sum_{n=1}^{N_{DGUs}}\zeta_{n}-\sum_{n_{b}=1}^{N_{b}}\zeta L_{b}=\sum_{n=1}^{N_{bus}}\zeta_{d}, \end{array}$$

Here, $\beta_{P}S$ and $\zeta_{PS}$ denote the active and reactive powers induced from the PS, $\beta_{n}$ and $\zeta_{n}$ are DGs generated active and reactive powers, active and reactive PLs for all branches, ’*b*’ is represented by $PL_{b}$ and $\zeta L_{b}$, the parameters $N_{DGs}$, $N_{b}$ and $N_{bus}$ are used for the quantity of DGs, branches and nodes in the proposed model. Correspondingly, the constraints of the power balance based on each node [11,12,17,24] are estimated as

(6) $$\begin{array}{c} \beta_{n}-\beta_{d}-V_{n}\sum_{n=1}^{N_{b}}V_{i}\left(G_{ni}cos\psi_{ni}+\beta_{ni}sin\psi_{ni}\right), \end{array}$$

(7) $$\begin{array}{c} \zeta_{n}-\zeta_{d}+\zeta_{c}-V_{n}\sum_{n=1}^{N_{b}}V_{i}\left(G_{ni}cos\psi_{ni}+\beta_{ni}sin\psi_{ni}\right), \end{array}$$

where $G_{ni}$ and $B_{ni}$ are the conductance and susceptance among *n* and *i* nodes, capacitive or inductive power levels are denoted by $\zeta_{c}$, $V_{n}$ and $V_{n}$ are the voltage parameters at *i* and *n* nodes and $\psi_{ni}$ describes the impedance angle between nodes *n* and *i*.

The active and reactive power levels of the DGs are laid between minimum and maximum limits [13,25], which are given as

(8) $$\begin{array}{c} \beta_{n,min}\leq \beta_{n}\leq \beta_{n,max}, \end{array}$$

(9) $$\begin{array}{c} \zeta_{n,min}\leq \zeta_{n}\leq \zeta_{n,max}, \end{array}$$

In addition to that, the node voltage in DS must be limited in the range of 0.95 to 1.05 [8,26] and is written as

(10) $$\begin{array}{c} V_{n,min}\leq V_{n}\leq V_{n,max}, \end{array}$$

In addition, it must be noted that the size of the thermal capacity branches is less than maximum thermal capacity [15,16], which is measured as

(11) $$\begin{array}{c} I_{b}\leq maxI_{b}, \end{array}$$

where $I_{b}$ presents the branch current. Secondly, the FCL size must exist between higher and lower ranges [27,28] and is shown as

(12) $$\begin{array}{c} min(SFCL)\leq SFCL\leq max(SFCL), \end{array}$$

### Types of DG Units for Distribution Networks

Four types are elaborated for DGs to deliver real and reactive powers, which are presented as follows.

Type 1: In this type, active and reactive powers are capable by DGs.
Type 2: Active power is managed by DGs only with a unity power factor, such as micro-turbines.
Type 3: Reactive power is controlled by DGs, such as a synchronous compensator.
Type 4: Consumption of reactive power with an injection of active power is a capability of DGs. This type includes a fixed-speed squirrel cage induction generator.

## 3. Methodology for Proper Allocation of DGs and FCLs

The key goal of this model is to treat issues of choosing the best placement for DGs and FCLs. The ICOA technique is presented for this purpose, which determines the population with $N_{packs}$ number of packs, where each pack consists of a number of coyotes $N_{coyote}$. Every coyote shows the best solution of placement for DGs and FCLs, including its social condition $C_{so}$ as decision variable $d_{v}$. The $d_{v}$ denotes the position of DGs and FCLs with active and reactive powers. Furthermore, the $C_{so}$ is calculated as

(13) $$\begin{array}{c} C_{so}^{packet,t}=\vec{j}=\left(j_{1},j_{2}...,j_{n}\right), \end{array}$$

The formulation of the initial coyote social conditions are presented as

(14) $$\begin{array}{c} C_{so,D,x}^{packet,t}=lb_{x}+r_{x}.\left(ub_{x}-lb_{x}\right), \end{array}$$

Here, *x* presents each $d_{v}$. lb is the lower bound and ub is the upper bound of the *x*th dv, and *r* shows a random number between 0 and 1. In order to minimize the power loss and fault current levels, a Fuzzy-Based Multi-Objective (FBMO) methodology is presented. Hence, the behavior of the coyote is adopted by the fitness function $F_{fit}$ and defined as

(15) $$\begin{array}{c} \xi_{FF}^{i,g}=\Gamma_{OF}^{i,g}+\chi_{PF}\sum_{n=1}^{N}{\left(\Omega_{PT}^{i,g,m}\right)}^{2}, \end{array}$$

Here, $\Omega_{PT}$ is used to violate the *m*th constraint in terms of *i*th solution for *g*th solution. The solution of the best $\xi_{FF}$ in each group is known as the best local solution, which is described by $LC_{best,g}$.

For the purpose of updating the social condition for the coyote in each group, updated solutions exist in COA around old solutions for two distances in terms of $LC_{best,g}$, which are (1) middle solution and (2) picked solution. These solutions are defined as

(16) $$\begin{array}{c} LC_{best,i,g}^{new}=LC_{best,t,h}+\gamma \left(LC_{best,g}-LC_{1,g}\right)\\ +\gamma (LC_{mid,g}-LC_{2,g}), \end{array}$$

where $LC_{1,g}$ and $LC_{2,g}$ are the picked solutions, and $LC_{mid,g}$ is the middle solution attained from *g*. The decision from middle solution is further elaborated as

(17) $$\begin{array}{c} LC_{mid,g}=\left\{\begin{array}{cc} \vartheta_{w,mid1} & \mathrm{if}\;\;N_{LC}\;\mathrm{is}\;\mathrm{odd},\\ \vartheta_{w,mid2}, & \mathrm{else},\forall w=1,2...N_{d\mu }, \end{array}\right. \end{array}$$

where $\vartheta_{w,mid1}$ and $\vartheta_{w,mid2}$ are the decision variables of $w_{th}$ numbers for odd and another two conditions. Moreover, each decision variable is positioned in descending order.

In order to select the performance procedure, each coyote *i* is related to old and new conditions, which correspond to old and new solutions, respectively. The $\xi_{FF}^{i,g}$ and $\xi_{FF}^{new}$ are considered for the quality of these two social conditions; thus, the following rules are used to retain a single social condition for each *i*th coyote.

(18) $$\begin{array}{c} LC_{i,g}=\left\{\begin{array}{cc} LC_{i,g}^{new} & \mathrm{if}\xi_{FF}^{new,i,g}\leq \xi_{FF}^{i,g},\\ LC_{i,g}, & \mathrm{else}, \end{array}\right. \end{array}$$

(19) $$\begin{array}{c} \xi_{FF}^{i,g}=\left\{\begin{array}{cc} \xi_{FF}^{new,i,g} & \mathrm{if}\xi_{FF}^{new,i,g}\leq \xi_{FF}^{i,g},\\ \xi_{FF}^{i,g}, & \mathrm{else}, \end{array}\right. \end{array}$$

From Equations (18) and (19), it is observed that ICOA produces two generations in every iteration. The produced solutions in the first generation in all groups are newly upgraded and the second generation includes only one upgraded solution for each group. Thus, these newly generated solutions in an iteration are measured as

(20) $$\begin{array}{c} N_{co}\times N_{g}+1\times N_{g}=N_{pop}+N_{g}, \end{array}$$

### Novelties and Contribution of the Proposed ICOA

The Coyote Optimize Algorithm (COA) has been broadly applied for streamlining issues in a wide range of fields. In [29], the authors have applied COA to limit the gas utilization of turbines in consolidated cycle power plants in Brazil. The proposed arrangement completely fulfilled the physical limits of the turbine and pollution emission regulations. The COA technique shows prevalence over different strategies like Artificial Bee Colony (ABC), Binary Switching Algorithm (BSA), Self-adaptive Differential Evolution (SaDE), Genetic Whale Optimization Algorithm (GWOA), Symbiotic Organism Search (SOS), and PSO. In [10], the authors were effective in applying COA to track down the fundamental elements of three diodes in photovoltaic modules. However, the acquired outcomes have not been contrasted with different strategies. In [30], the financial dispatch issue with nuclear energy stations and wind turbines was tackled by COA, GA, and PSO. The acquired outcomes from two examined frameworks showed that the COA arrived at a preferable arrangement over GA and PSO, yet there was no exhibit about the quicker speed of COA since settings of populace and cycles were overlooked. Another example of improvements to the shortcomings of COA is shown in [31]. COA has been applied for managing issues with block coordinating, and the consequences of COA were contrasted with Enhanced Gray Wolf Algorithm (EGWA) and different strategies. A discussion of results in [32] showed that COA was less compelling than EGWA and a portion of different techniques like Black Hole Algorithm (BHA), Gray Wolf Algorithm (GWA), and PSO. ICOA is an improved metaheuristic calculation achieved by performing two fundamental adjustments to the first Coyote Streamlining Calculation (COA). The two proposed adjustments aim at improving the ideal arrangement quality found by the original and second stages in the COA strategy. The COA technique was created in 2018 [33], dependent on the normal practices of coyotes. Every coyote is portrayed by two fundamental components, social condition and nature of the social condition in which social condition is related to the ideal arrangement, and the nature of the social condition is compared to the wellness capacity of the arrangement. The local coyote area is partitioned into Ng little coyote gatherings, and there are Nco coyotes in each gathering. The COA strategy produces two new arrangement stages for every cycle in which the original produces Nco new answers for each gathering and the subsequent stage produces Ng new answers for the entire coyote local area. Consequently, the absolute number of new arrangements created in the COA technique is (Nco × Ng + Ng) arrangements in which Nco × Ng is equivalent to the populace (Npop). Along these lines, (Nco × Ng + Ng) is equivalent to (Npop + Ng). The number of new answers for every stage can show that the original affects the last arrangement quality since it produces Nco-times new arrangements of the subsequent stage. Be that as it may, COA is adapting to the low execution of the original since it utilizes a middle answer for creating an updated advanced size; in the meantime, the solid mark of the middle arrangement is only to deliver a variety of arrangements, and it does not have the potential to produce a promising updated, advanced size. In the subsequent stage, COA produces one new answer for each group by utilizing a randomization factor. The new arrangements are shaped by randomization, and control factors in the arrangement can be either taken from the current arrangements or haphazardly created inside lower and upper limits. In any case, it should utilize one out of three unique choices for each new control variable in the arrangement, and two control boundaries should be resolved for the choices. Unmistakably, the subsequent stage relies on randomization, and the time has come to enforce two control boundaries. Therefore, in the ICOA strategy, we propose two alterations to the first and the second new arrangement stages. In the original, the arrangement is replaced with the best arrangement yet, with a plan to improve the nature of recently delivered arrangements and lessen reenactment time. In the subsequent alteration, each gathering produces one new arrangement based on the best arrangement by utilizing a few updated advancements reliant upon the number of pair arrangements, which are combined. The subsequent adjustment can improve the adequacy of the neighborhood search and discover one promising answer for each gathering. ICOA has some benefits over COA, for example, (i) decreasing computational time, (ii) improving solution quality, and (iii) arriving at better stability in the pursuit interaction.

## 4. Results and Discussion

**Figure 1:**
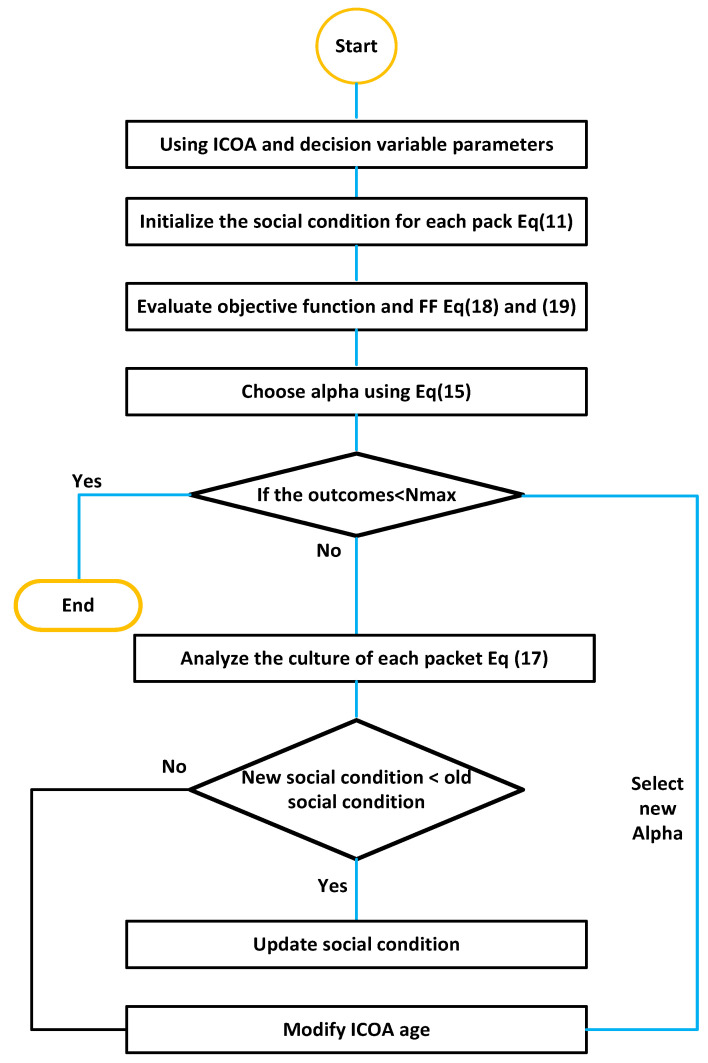
Flow chart of the proposed model for placement of DGs and FCL.

IEEE-28 bus systems are designed to analyze the optimum location of DGs and FCLs using ICOA. The flow chart for analyzing optimum allocation for DGs and FCLs is presented in Figure 1. The proposed model is discussed in terms of three cases, listed as

Case 1: DGs working at a unity power factor and related to Type 3.
Case 2: The power factor is kept constant for DGs, related to Type 1.
Case 3: Controllable power factor technique is used for DGs.

In Case 1, where DGs are working with the unity power factor, the ICOA is employed, and the outcomes are presented in Table 1. It is depicted from Table 1 that ICOA has chosen the best location for DGs at buses 12, 22 and 28, including 1.0834, 1.077 and 0.9244 MW sizes, respectively. In addition, the attained outcomes are compared with Firework Algorithms (FWA), Bacterial Foraging Optimization Algorithm (BFOA), Harmony Search Algorithm (HSA), Taguchi Algorithm (TA)-based approach and Water Cycle Algorithm (WCA).

Table 1 shows the efficient results of ICOA over other used algorithms. Similarly, results taken based on Case 2 and Case 3 are explained in Table 2. The outcomes of ICOA in Case 2 show that the losses are much less than Case 1. Furthermore, the best position of DGs is selected by using Case 3, as shown in Table 2. Thus, power losses are reduced and minimum voltage is improved compared to the initial case.

**Figure 2:**
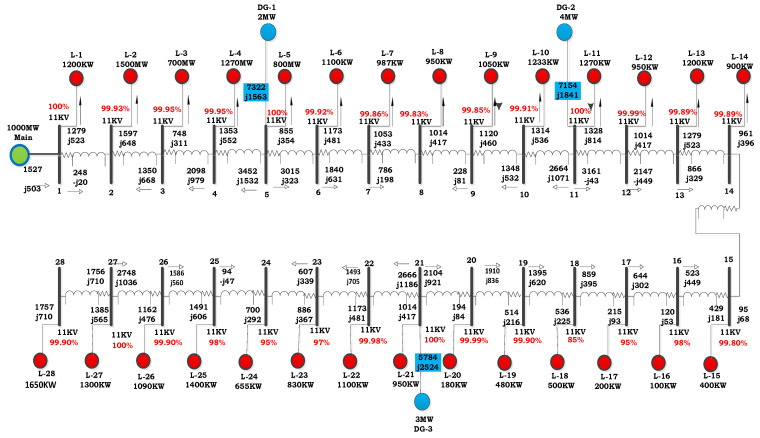
IEEE-28 bus Proposed model.

The proposed model was designed in ETAP, as shown in Figure 2. The selected proposed design is used to demonstrate load flow analysis and ETAP. ETAP is an important simulation tool for learning. The features of ETAP provide a reliable and easy design for three-phase and single-phase AC/DC networks with bus bars and all their components; for example, grounding and instrumentation components with selected values and parameters of various systems can be entered, such as dynamic and static data for modeling.

The analysis of load flow needs input data, which consists of nominal values, the impedance of generators, lines and DGs, and other values, and these are shown in Figure 2. Libraries (database) of ETAP provide a whole set of validated and verified data from instrument manufacturers. The process of a whole simulation has become more convenient and efficient, and there is no need for extra data, as the typical values of specific parts of the system can be used for analysis.

Table 3 displays the simulation outcomes of COA and ICOA for constant PF of DGUs and different PFs of DGUs. In the case of constant PF, COA selects DGUs with sized 0.71245, 1.0379 and 1.2004 MW to be placed at buses 14, 24, and 27, and ICOA picks DGUs with size 2, 4 and 3 MW to be set at buses 5, 11and 21, respectively. These fallouts demonstrate that the power losses are considerably less than the initial case, as well as COA, where the minimum VP is significantly improved.

ICOA Technique for Allocation of DGs: The planned ICOA is chosen for finding the simultaneous site and sizing of DGUs in a single-stage method. The gained outcomes are compared to the bi-stage method. In Table 4, the ICOA illustrates better efficiency as compared to the other two. Furthermore, a higher PL reduction of 42.132 kW was achieved, as shown in Table 4. Furthermore, the ICOA has a mean time of 1.5 s. The engaged ICOA proves high efficiency in determining the lowest power losses as compared to other approaches, as shown in Table 1 and Table 3. However, the ICOA is run for 28 times for the cases when DGs has constant and different PF, and the minimum, average, maximum and standard deviation (Std) are assessed. Comparisons with the most well-known method, PSO, are also presented in Table 5. These outcomes show the ICOA competence of a single stage for choosing the optimum DGUs sizes. Consequently, the gained outcomes proclaim the planned technique has great robustness.

**Figure 3:**
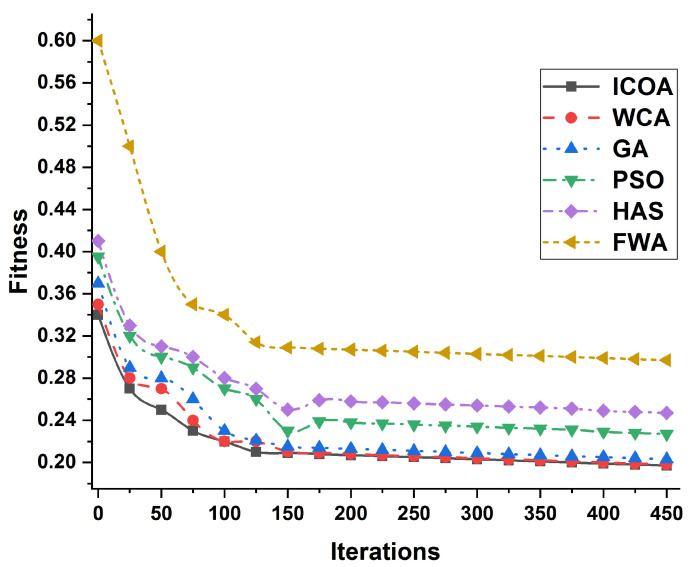
Comparison of different algorithms with ICOA for the proposed IEEE-28bus model.

The performance of the proposed model is also analyzed using graphical analysis, where Figure 3 explores the results among different iterations against fitness. Several modern algorithms like Water Cycle Algorithm (WCA), Genetic Algorithm (GA), Particle Swarm Optimization (PSO), Harmony Search Algorithm (HSA) and Fireworks Algorithm (FWA) are compared with the proposed ICOA schemes. The outcomes of the proposed model in Figure 3 clarify that the fitness range of ICOA is much better than the other algorithms used.

**Figure 4:**
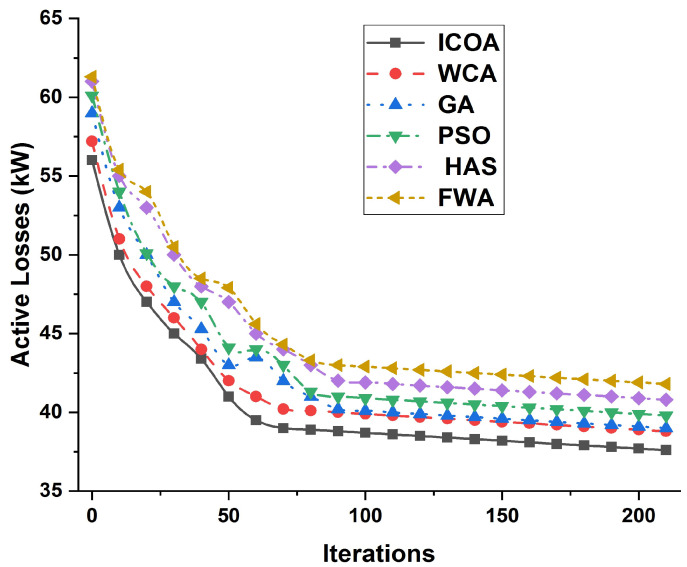
Active power losses for different iterations to compare ICOA, WCA, GA, PSO, HAS and FWA algorithms.

Figure 4 explains the comparison of ICOA, WCA, GA, PSO, HAS and FWA algorithms in terms of active losses for different iterations, which shows that the efficiency of the ICOA is more supportive than the currently employed algorithms.

**Figure 5:**
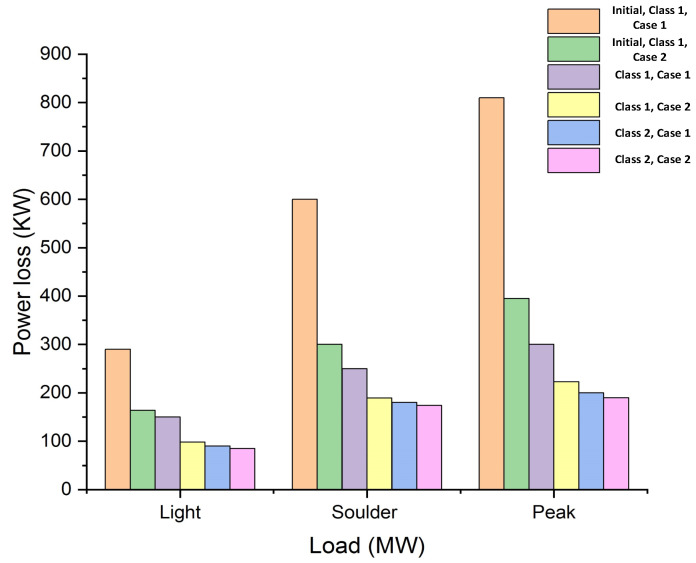
Graphical results analysis for light, shoulder and peak hour loads and power loss for the initial case, Class 1, Class 2, Case 1 and Case 2.

The results of power losses as a function of light, shoulder and peak loads are investigated in Figure 5 for initial cases and the proposed ICOA cases, including class 1 and class 2 conditions. It is depicted from the results that the initial case contains huge losses and, thus, is limited in fulfilling electricity demands. On the other hand, the outcomes of the ICOA-based system generate low losses even at peak loads.

**Figure 6:**
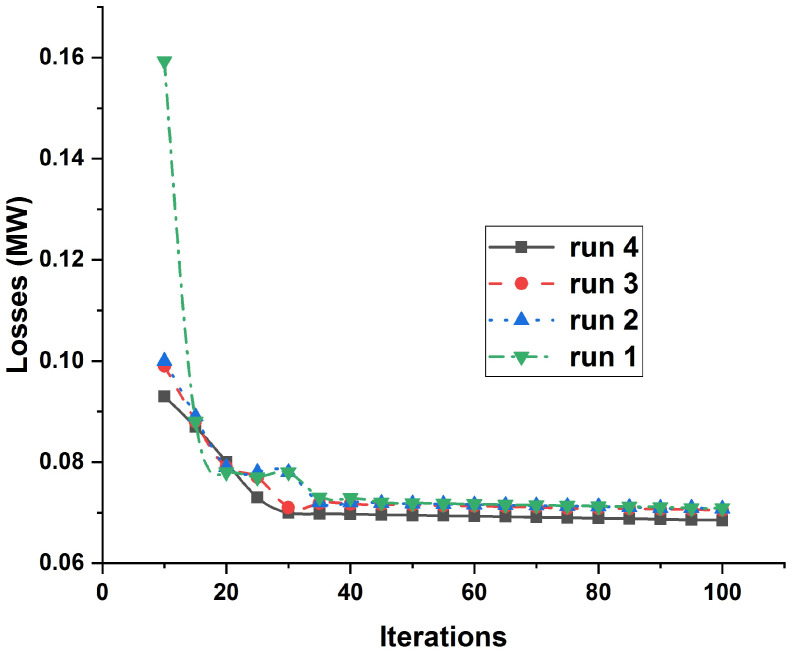
Convergence rate of the proposed ICOA for different iterations and runs against power losses.

The employed ICOA is tested at different runs for case 1 and class 1, as declared in Figure 6, depicting high robustness as compared to other algorithms mentioned in Table 4. Furthermore, optimal allocation of DGUs is revealed by applying ICOA, as presented in Table 5.

**Figure 7:**
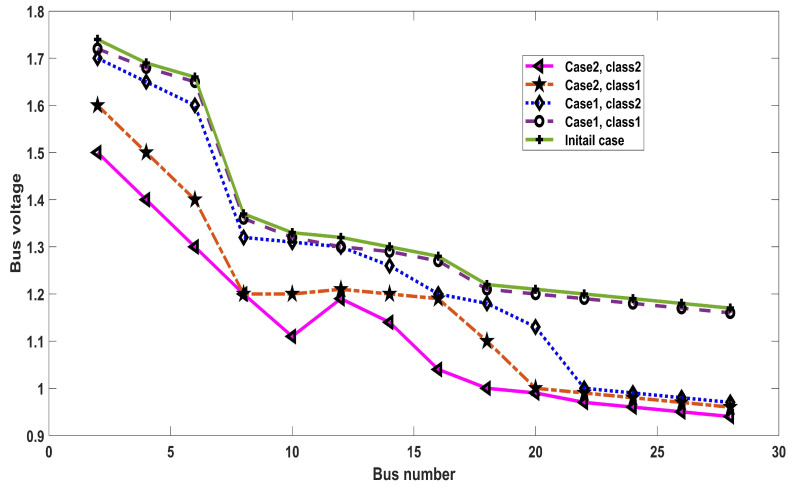
Analysis of bus voltage and number at initial and proposed cases.

**Figure 8:**
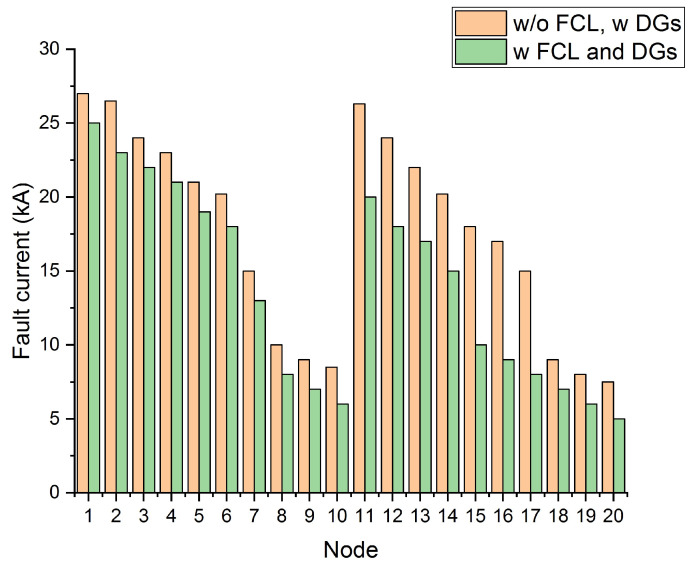
Comparison of outcomes with FCL and without FCL.

The simulation analysis among bus voltage and numbers is evaluated in Figure 7 for the initial and proposed cases, presenting fruitful outcomes of the proposed ICOA for both cases and classes. Figure 8 depicts the fault current for each FCL size. The results are compared with FCL and without FCL, using short circuit analysis. It is clarified from Figure 8 that the inclusion of FCL decreased the fault current levels.

## 5. Conclusions

The balance of electricity demands and power losses with proper allocation of DGs are highlighted issues in installed power systems. Therefore, to enhance the voltage profile and to measure better placement of DGs in the distribution system, the ICOA algorithm is analyzed in this paper for an IEEE 28-bus system. The major constraints and their solutions are also addressed analytically, which concludes that by compensating the power and voltage constraints, a stable distribution network cannot be designed. The proposed ICOA technique for allocating and sizing DGs is explored in different steps to show how fitness functions are defined as solutions to the generated issues. In addition, the results of the proposed model are declared in tabular and graphical forms, using different cases and classes. It was found that, when compared, the proposed ICOA outperforms the other optimizers. The optimization placement of DG units is proposed, correlated with FCLs in single-phase as compared to multi-phase. To reduce power losses and fault current levels, an improved coyote algorithm (ICOA) methodology is used. The simulation results present that the ICOA mechanism helps in choosing the best position for DGs and FCLs in the distribution network. Using the ICOA procedure, the proposed setup is able to achieve acceptable outcomes and minimize power losses and faulty current levels. It is found that a huge amount of power losses are reduced by increasing the voltage profile. Hence, the issues of optimum placement of DGUs in distribution networks and decreasing economical and technical issues may be improved by considering the proposed ICOA model.

## Figures and Tables

**Table 1:** Using Case 1 for optimum allocation of DGs for 28 bus system.

Mechanism	KW (Losses)	DG Size/Placement	Min Voltage (P.U) Bus
TA [8]	89.214	0.5897(14), 0.189(18), 1.0146(21)	0.968
FWA [16]	98.3	0.633(17), 0.09(18), 0.947(27)	0.964
HAS [15]	96.76	0.5724(17), 0.107(18), 1.0462(19)	0.967(24)
BFOA [27]	103.4	0.925(11), 0.863(16), 1.2(21)	0.98(25)
PSO [28]	105.35	1.1768(8), 0.9816(13), 0.8297(24)	0.98(21)
GA [1]	106.3	1.5(11), 0.4228(29), 1.0714(20)	0.981(25)
WCA [14]	72.9	0.8546(14), 1.1017(24), 1.181(29)	0.97(16)
ICOA	40.35	2(5), 4(11), 3(21)	0.988(8)

**Table 2:** Optimum placement of DGs in Case 2 and Case 3.

	KW (Losses)	DG Size/Placement	Min Voltage (P.U) Bus	Power Factor
Initial	213.78	-	0.99(17)	-
Case 2	17.54	0.8232(12), 1.1397(22), 1.12(26)	0.994(7)	0.84–0.85
Case 3	12.8	0.837(12), 1.124(22), 1.07(26)	0.994(7)	0.75–0.86

**Table 3:** Allocation of DGu and FCL using the proposed ICOA-base FBMO.

Framework	Losses (KW)	DG Size/Placement
ETAP-Bi Stage	44.341	5.874(6), 8(16), 5.074(22)
COA (One Stage)	44.815	7.783(16), 5.6056(22), 5.8189(9)
ICOA (One Stage)	42.132	2(5), 4(11), 3(21)

**Table 4:** Comparison among PSO, COA and ICOA for DGu placement and sizing.

Network	28-Bus System		
Method	PSO	COA	ICOA
Min(MW)	0.817	0.0715	0.0690
Mean(MW)	0.0758	0.0739	0.0701
Max(MW)	0.0723	0.0794	0.0694
Std	0.0026	0.0020	0.0013

**Table 5:** Allocation of DGu for 28-Bus System.

		Losses (KW)	DG Size (MW) and Location	DGu Power Factor	Min. Voltage Profile % (Bus)
Initial		4871.6	–	–	80.34(28)
COA	Different power factor	14.43	0.71245(14), 1.0379(24), 1.2004(27)	0.85, 0.85, 0.85	99.2(8)
	Constant Power Factor	11.7	0.7294(14), 1.0538(24), 1.0953(27)	0.8951, 0.9024, 0.7302	99.2(8)
ICOA	Different power factor	15.12	2(5), 4(11), 3(21)	0.8951, 0.9024, 0.7302	99.12(7)
	Constant Power Factor	10.34	2(5), 4(11), 3(21)	0.85, 0.85, 0.85	99.12(7)
